# A monoclonal antibody targeting ErbB2 domain III inhibits ErbB2 signaling and suppresses the growth of ErbB2-overexpressing breast tumors

**DOI:** 10.1038/oncsis.2016.25

**Published:** 2016-03-21

**Authors:** Y Meng, L Zheng, Y Yang, H Wang, J Dong, C Wang, Y Zhang, X Yu, L Wang, T Xia, D Zhang, Y Guo, B Li

**Affiliations:** 1 School of Medicine, Nankai University, Tianjin, People's Republic of China; 2 International Joint Cancer Institute, the Second Military Medical University, Shanghai, People's Republic of China; 3 Department of Vascular Surgery, Changhai Hospital, Second Military Medical University, Shanghai, People's Republic of China; 4 State Key Laboratory of Antibody Medicine and Targeted Therapy, Shanghai, People's Republic of China

## Abstract

The anti-ErbB2 antibodies trastuzumab and pertuzumab in combination have recently been approved for the treatment of patients with ErbB2-positive metastatic breast cancer. Pertuzumab, which binds to ErbB2 near the center of domain II, and trastuzumab, which binds to the juxtamembrane region of ErbB2 domain IV, directly interfere with domain II- and domain IV-mediated heterodimerization contacts, respectively. In this study, we report a novel anti-ErbB2 antibody, 3E10, which binds to an epitope in domain III that appears to be located opposite to the dimerization interfaces in domain II and domain IV of ErbB2. Our data show that the 3E10 antibody inhibits ErbB2 heterodimerization via a mechanism that strikingly differs from trastuzumab and pertuzumab. It could be speculated that the 3E10 antibody may affect ErbB2 heterodimerization by causing major conformational changes of ErbB2. Furthermore, 3E10 provides synergistic inhibition of ErbB2 heterodimerization and signaling in combination with either trastuzumab or pertuzumab. The combination of these three anti-ErbB2 antibodies that have complementary mechanisms of action appears to be an extremely potent ErbB2 heterodimerization blocker. Compared with trastuzumab plus pertuzumab, the combination of trastuzumab, pertuzumab and 3E10 provides a more potent blockade of ErbB2 signaling. Consistent with this, trastuzumab plus pertuzumab plus 3E10 results in greater *in vitro* and *in vivo* antitumor activity in ErbB2-overexpressing breast tumor models, suggesting its potential use for treating ErbB2-overexpressing breast cancer.

## Introduction

Overexpression of human epidermal growth factor receptor-2 (HER2 or ErbB2), a member of the ErbB family of receptor tyrosine kinases, is found in 25–30% of human breast cancers, and correlates with more aggressive tumors and a poorer prognosis.^1, 2^ Trastuzumab, a humanized monoclonal antibody (mAb) directed against ErbB2, is the first anti-ErbB2 treatment approved for clinical use for patients with ErbB2-overexpressing metastatic breast cancer.^3^ However, the objective response rate for trastuzumab monotherapy is only 26%, and only 6% of patients experience a complete response.^4^ Thus, there is an urgent need to improve ErbB2-directed therapy.

Although no specific ligand for ErbB2 has been identified, ErbB2 is the preferred heterodimerization partner of the ErbB family.^5^ ErbB2 forms heterodimers with both ligand-free and ligand-bound forms of the other three ErbB family members (EGFR, ErbB3 and ErbB4), which activates ErbB receptors and downstream MAPK and AKT signaling pathways, thereby promoting cell proliferation and survival.^3, 6, 7, 8, 9, 10, 11^ Previous studies have demonstrated that trastuzumab is not capable of inhibiting signaling by ligand-induced ErbB2/ErbB3 heterodimer.^12^ In contrast, pertuzumab, another ErbB2-specific humanized antibody that binds to a distinct epitope from trastuzumab,^13, 14, 15^ efficiently inhibits ligand-mediated ErbB2/ErbB3 complex formation.^12^ Interestingly, in the absence of ErbB3 ligand, the abilities of these two antibodies to inhibit ErbB2/ErbB3 heterodimer formation are reversed.^8^ The combination of these two anti-ErbB2 antibodies that have complementary mechanisms of action synergistically inhibits the *in vitro* and *in vivo* growth of ErbB2-overexpressing breast cancer cell lines.^16, 17^ Recently, trastuzumab plus pertuzumab has been approved for the treatment of patients with ErbB2-overexpressing metastatic breast cancer.

ErbB2 is proposed to heterodimerize with ligand-bound ErbBs using a largely domain II-mediated dimerization interface.^18^ In contrast, ErbB2 heterodimerization with ligand-free ErbBs may be mainly involved in domain IV contacts.^8^ Pertuzumab, which binds to ErbB2 near the center of domain II,^15^ and trastuzumab, which binds to the juxtamembrane region of domain IV,^14^ directly interfere with domain II- and domain IV-mediated heterodimerization contacts, respectively. Here we report an ErbB2 domain III-specific antibody, which inhibits heterodimerization via a mechanism that strikingly differs from trastuzumab and pertuzumab. Moreover, the ErbB2 domain III-specific antibody provides synergistic inhibition of ErbB2 in combination with either trastuzumab or pertuzumab. The combination of the three anti-ErbB2 antibodies that have different mechanisms of action exhibits superior efficacy over the combination of trastuzumab and pertuzumab.

## Results

### An ErbB2 domain III-specific antibody inhibits ErbB2 heterodimerization and signaling

**Figure 1:**
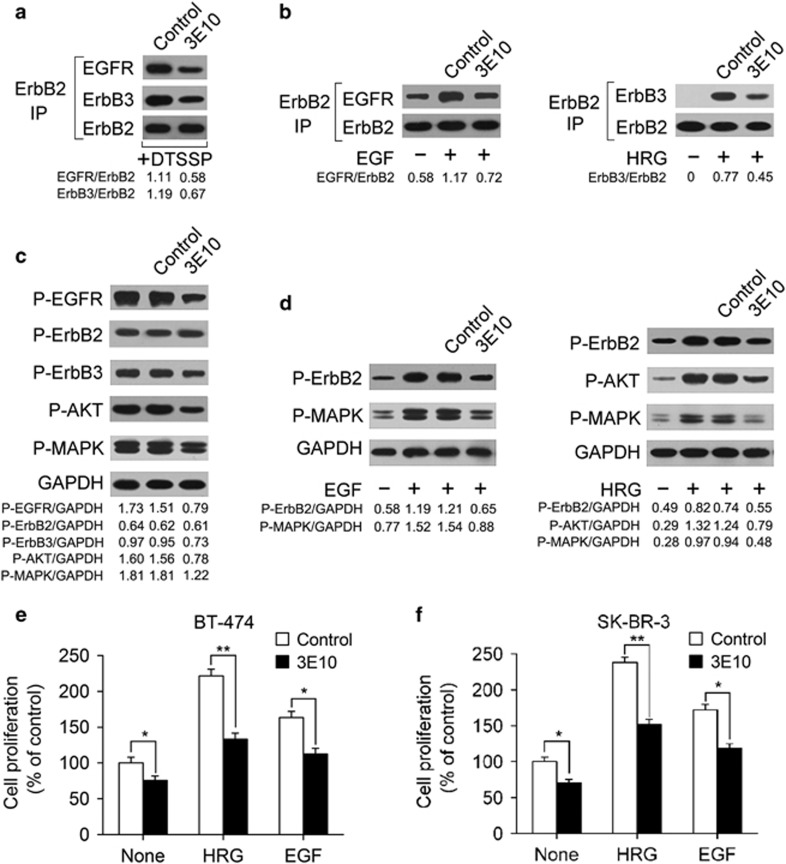
The 3E10 antibody inhibits ErbB2 signaling and cell growth in ErbB2-overexpressing breast cancer cell lines. (**a**) Coimmunoprecipitation assay examining the ability of 100 nM of control IgG or 3E10 to disrupt the formation of ligand-independent ErbB2-containing heterodimers in BT-474 cells. (**b**) Coimmunoprecipitation assay detecting EGF-induced ErbB2/EGFR and HRG-induced ErbB2/ErbB3 heterodimerization in BT-474 cells pretreated with 100 nM of control IgG or 3E10. (**c**) Immunoblots assessing ErbB2 signaling in BT-474 cells upon treatment with 100 nM of control IgG or 3E10 in the absence of ErbB ligand. (**d**) Immunoblots evaluating the effects of 100 nM of control IgG or 3E10 pretreatment on EGF- or HRG-activated ErbB2 signaling in BT-474 cells. (**e** and **f**) MTS assay examining the effects of 100 nM of control IgG or 3E10 on breast cancer cell proliferation in the absence or presence of ErbB ligand (EGF or HRG). Results are shown as percentage of control cell proliferation. Error bars, s.d. *P<0.05, **P<0.001.

Two ErbB2-overexpressing breast cancer cell lines, BT-474 and SK-BR-3, were used in this study. We generated a panel of mouse mAbs specific for ErbB2 and determined their ability to block ErbB2 heterodimerization in BT-474 cells. Surprisingly, we found that an ErbB2-specific mouse mAb (IgG2a,κ), denoted as 3E10, effectively inhibited both ligand-independent and -dependent ErbB2 heterodimerization (Figures 1a and b). We next examined the inhibitory effects of 3E10 treatment on the activation of ErbB2 and downstream MAPK and AKT signaling pathways in BT-474 cells. The results showed that 3E10 inhibited both ligand-independent and ligand-induced ErbB2 signaling (Figures 1c and d). Moreover, we evaluated the ability of 3E10 to inhibit the *in vitro* proliferation of BT-474 and SK-BR-3 cell lines. The data indicated that 3E10 was effective in suppressing breast cancer cell proliferation in the absence of ErbB ligand (Figure 1e and f). The 3E10 antibody also significantly inhibited the *in vitro* growth of HRG- and EGF-stimulated breast cancer cells (Figure 1e and f).

### Mapping of 3E10-specific epitope on the extracellular domain of ErbB2

**Figure 2:**
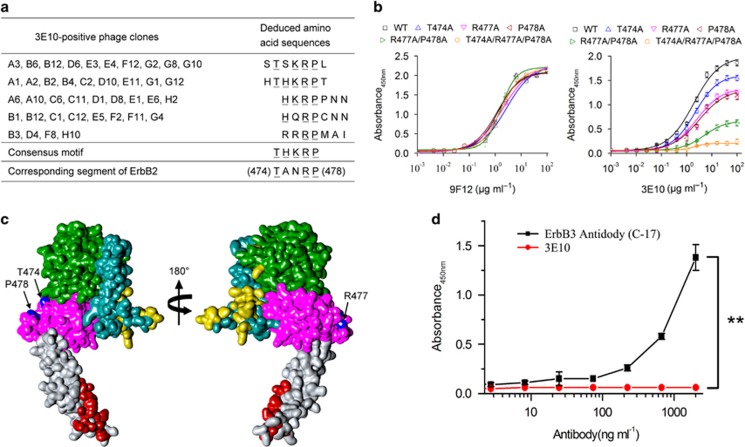
Mapping of 3E10-specific epitope on the extracellular domain of ErbB2. (**a**) Amino-acid sequences of the insert from 3E10-positive phage clones. Sequences were aligned for the consensus motif, which is indicated by underlined letters. (**b**) Effect of alanine substitutions on 3E10 binding to ErbB2. Data are expressed as means±s.d. (**c**) Surface representation of the extracellular domain of ErbB2 (PDB accession code 1N8Z). Domains I, II, III and IV are green, dark cyan, magenta and gray, respectively. The ErbB2 residues within 5 Å of trastuzumab (PDB accession code 1N8Z) and pertuzumab (PDB accession code 1S78) are colored red and yellow, respectively. The 3E10 epitope residues, T474, R477 and P478, are colored blue. (**d**) 3E10 did not cross-react with ErbB3. Different concentrations of the 3E10 antibody were added to 96-well plates precoated with 3 μg/ml of recombinant human ErbB3/Her3 Fc chimera Protein (R&D Systems), followed by incubation at 37 °C for 1 h. After washing, horseradish peroxidase-labeled goat anti-mouse IgG H&L (Abcan) was added and the plates were further incubated for 1 h at 37 °C. Finally, 3,3′,5,5′-tetramethylbenzidine (TMB) was added as a substrate and the absorbance was read at 450 nm. As a positive control, different concentrations of horseradish peroxidase-labeled ErbB-3 antibody (C-17) (sc-285; Santa Cruz Biotechnology) were added to 96-well plates precoated with 3 μg/ml of recombinant human ErbB3/Her3 Fc chimera Protein, followed by incubation at 37 °C for 1 h. After washing, TMB was added as a substrate and the absorbance was read at 450 nm.

To identify the peptides recognized by 3E10, phage clones were isolated by panning the PhD-7 phage display peptide library with 3E10. Three rounds of selection were performed and at each round, the library was precleared on a control mouse IgG2a,κ antibody. After the third round of panning, the binding of the isolated phage clones to 3E10 was determined in an enzyme-linked immunosorbent assay (ELISA). Sequence analysis of 3E10-positive phage clones identified five distinct amino-acid sequences (Figure 2a). Alignment of these sequences resulted in the consensus motif THKRP, which could be aligned with the ^474^T—R^477^P^478^ sequence located at the extracellular domain III of ErbB2 (Figure 2a). To prove that the ^474^T—R^477^P^478^ sequence within ErbB2 is the epitope recognized by 3E10, alanine substitutions were introduced into ErbB2-ECD at residues T474, R477 and P478, and the binding of 3E10 to these ErbB2-ECD mutants was measured by ELISA. The results showed that alanine substitution in any one of residues T474, R477 and P478 significantly reduced 3E10 binding activity for ErbB2 (Figure 2b). Double alanine substitutions at positions 477 and 478 further reduced the binding activity, and triple alanine substitutions at positions 474, 477 and 478 almost totally abolished 3E10 binding to ErbB2 (Figure 2b). In contrast, the binding of 9F12, another mouse anti-ErbB2 mAb that does not compete with 3E10, to these ErbB2-ECD mutants was approximately the same as to wild-type ErbB2-ECD (Figure 2b). These data demonstrate that the ^474^T—R^477^P^478^ sequence within ErbB2 is the 3E10 epitope. The 3E10 epitope appeared to be located opposite to the dimerization interfaces in domains II and IV (Figure 2c), implying that 3E10 did not directly disrupt the interaction of ErbB2 with other ErbBs. Therefore, we speculate that the 3E10 antibody may exert the ErbB2 heterodimerization-blocking activity through causing major conformational changes of the ErbB2 molecule.

The ErbB2 epitope recognized by the 3E10 antibody is centered on the sequence ‘TANRP'. Within this sequence, amino acids R and P appear to be critical (Figure 2b). Interestingly, residues R and P, are also conserved in ErbB3 as well as the residue N. Therefore, three out of five amino acids of the indicated epitope are shared by both ErbB2 and ErbB3. Next, we investigated if the 3E10 antibody might exhibit some cross-reactivity toward ErbB3. The anti-ErbB-3 antibody (C-17) was used as a positive control. Our results showed that the C-17 antibody reacted with ErbB3 but the 3E10 antibody did not recognize ErbB3 (Figure 2d), suggesting that 3E10 did not cross-react with ErbB3.

### The 3E10 antibody provides synergistic inhibition of ErbB2 signaling in combination with antibodies directly blocking ErbB2 heterodimerization

**Figure 3:**
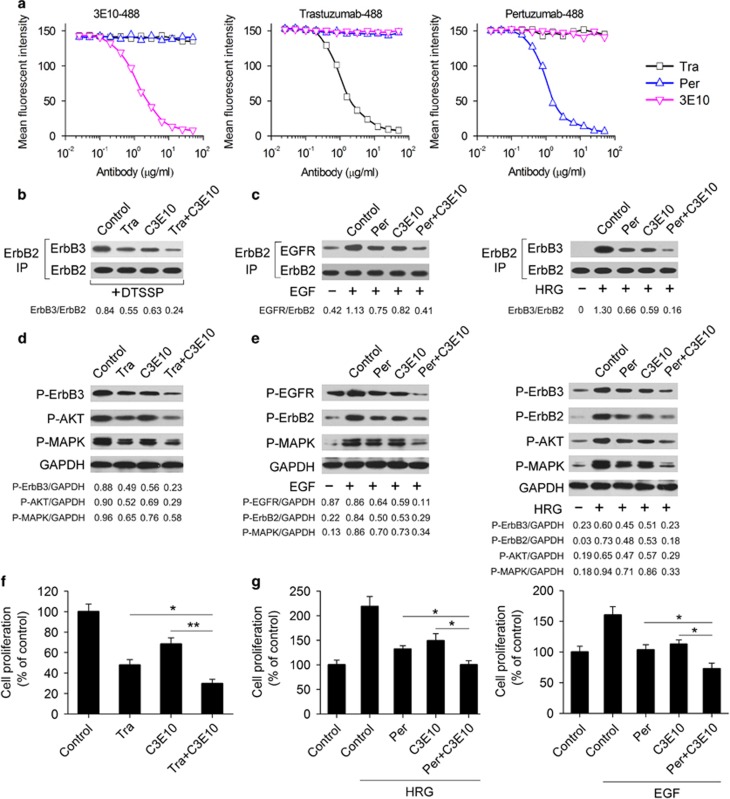
Combination of C3E10 with either trastuzumab or pertuzumab synergistically inhibits ErbB2 heterodimerization and signaling. (**a**) Competitive binding assay. 3E10, trastuzumab and pertuzumab were evaluated for their ability to compete with Alexa Fluor 488-labeled 3E10, Alexa Fluor 488-labeled trastuzumab or Alexa Fluor 488-labeled pertuzumab for binding to ErbB2-overexpressing BT-474 cells. (**b**) Coimmunoprecipitation assay comparing the ability of control IgG (10 μg/ml), trastuzumab (10 μg/ml), C3E10 (10 μg/ml) and trastuzumab plus C3E10 (5 μg/ml each) to disrupt ligand-independent ErbB2/ErbB3 heterodimer formation in BT-474 cells. (**c**) Coimmunoprecipitation assay detecting EGF-induced ErbB2/EGFR and HRG-induced ErbB2/ErbB3 heterodimerization in BT-474 cells pretreated with control IgG (10 μg/ml), pertuzumab (10 μg/ml), C3E10 (10 μg/ml) or pertuzumab plus C3E10 (5 μg/ml each). (**d**) Immunoblots examining ErbB2 signaling in BT-474 cells upon treatment with control IgG (10 μg/ml), trastuzumab (10 μg/ml), C3E10 (10 μg/ml) or trastuzumab plus C3E10 (5 μg/ml each) in the absence of ErbB ligand. (**e**) Immunoblots assessing the effects of control IgG (10 μg/ml), pertuzumab (10 μg/ml), C3E10 (10 μg/ml) or pertuzumab plus C3E10 (5 μg/ml each) pretreatment on EGF- or HRG-activated ErbB2 signaling in BT-474 cells. (**f**) MTS assay comparing the effects of control IgG (10 μg/ml), trastuzumab (10 μg/ml), C3E10 (10 μg/ml) and trastuzumab plus C3E10 (5 μg/ml each) on BT-474 cell proliferation in the absence of ErbB ligand. Results are shown as percentage of control cell proliferation. Error bars, s.d. *P<0.05, **P<0.001. (**g**) MTS assay assessing the effects of control IgG (10 μg/ml), pertuzumab (10 μg/ml), C3E10 (10 μg/ml) and pertuzumab plus C3E10 (5 μg/ml each) on BT-474 cell proliferation in the presence of HRG or EGF. Results are shown as percentage of control cell proliferation. Error bars, s.d. *P<0.05.

Next, we investigated if the 3E10 antibody could compete with trastuzumab or pertuzumab for binding to ErbB2-overexpressing BT-474 cells. Our data showed that 3E10 did not compete with either trastuzumab or pertuzumab (Figure 3a). The 3E10 antibody and trastuzumab (or pertuzumab) bind to distinct regions of ErbB2 and inhibit receptor activation by different mechanisms. This raises a question of possible synergistic inhibition of ErbB2 signaling by 3E10 and trastuzumab (or pertuzumab). Our data showed that the combination of C3E10 (a mouse/human chimeric IgG1/κ antibody derived from 3E10) and trastuzumab inhibited ErbB2 heterodimerization significantly more efficiently than either mAb alone (Figure 3b). Similar results were observed for C3E10 plus pertuzumab (Figure 3c). Consistently, C3E10 in combination with either trastuzumab or pertuzumab synergistically inhibited the phosphorylation of ErbB receptors and their downstream signaling molecules MAPK and AKT (Figures 3d and e), and the proliferation of BT-474 cells (Figures 3f and g).

### The combination of trastuzumab, pertuzumab and C3E10 has superior antitumor activity compared with trastuzumab plus pertuzumab

**Figure 4:**
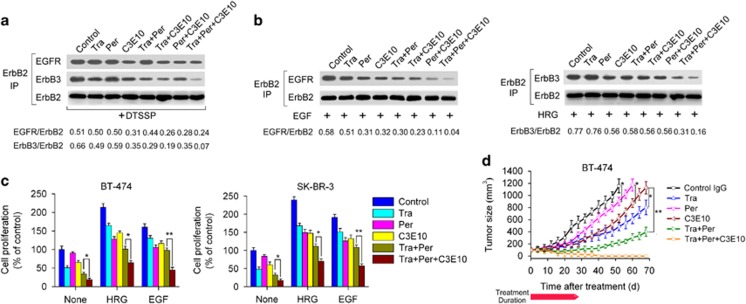
The combination of trastuzumab, pertuzumab and C3E10 potently blocks ErbB2 heterodimerization and inhibits breast cancer cell growth. (**a**) Coimmunoprecipitation assay examining the ability of 100 nM of control IgG, trastuzumab, pertuzumab, C3E10, trastuzumab plus pertuzumab, trastuzumab plus C3E10, pertuzumab plus C3E10 or trastuzumab plus pertuzumab plus C3E10 to disrupt the ligand-independent association of ErbB2 with EGFR or ErbB3 in BT-474 cells. (**b**) Coimmunoprecipitation assay assessing the effects of 100 nM of control IgG, trastuzumab, pertuzumab, C3E10, trastuzumab plus pertuzumab, trastuzumab plus C3E10, pertuzumab plus C3E10 or trastuzumab plus pertuzumab plus C3E10 pretreatment on EGF-induced ErbB2/EGFR and HRG-induced ErbB2/ErbB3 heterodimerization in BT-474 cells. (**c**) MTS assay evaluating the effects of recombinant anti-ErbB2 mAbs on breast cancer cell proliferation in the absence or presence of ErbB ligand (EGF or HRG). Cells were incubated with 100 nM of control IgG, trastuzumab, pertuzumab, C3E10, trastuzumab plus pertuzumab or trastuzumab plus pertuzumab plus C3E10 for 2 h, followed by the addition of ErbB ligands or not. Recombinant human EGF and HRG were added at a final concentration of 5 and 1 nM, respectively. After an additional 4-day incubation, cell proliferation was determined by MTS assay. Results are shown as percentage of control cell proliferation. Error bars, s.d. *P<0.05; **P<0.001. (**d**) Tumor volume of BT-474 breast tumor xenografts after treatment with control IgG (5 mg/kg), trastuzumab (5 mg/kg), pertuzumab (5 mg/kg), C3E10 (5 mg/kg), trastuzumab plus pertuzumab (5 mg/kg each) or trastuzumab plus pertuzumab plus C3E10 (5 mg/kg each). Treatments consisted of twice weekly intravenous injection of different anti-ErbB2 mAbs for four consecutive weeks. Data are shown as means±s.e.m. *P<0.05; **P<0.001; Mann–Whitney test.

We investigated the effects of the combination of C3E10 with trastuzumab and pertuzumab on ErbB2 heterodimerization (Figures 4a and b). Our results showed that this combination appeared to be a much more potent ErbB2 heterodimerization blocker than trastuzumab plus pertuzumab. Consistent with this, the three mAbs in combination had a significantly greater ability to inhibit the *in vitro* breast cancer cell proliferation compared with trastuzumab plus pertuzumab (Figure 4c). Next, the therapeutic efficacy of anti-ErbB2 mAbs was compared in nude mice bearing established BT-474 xenograft tumors. We demonstrated that trastuzumab plus pertuzumab plus C3E10 was more efficient in inhibition of BT-474 tumors than combinatorial treatment with trastuzumab and pertuzumab (Figure 4d). Importantly, all tumors were completely eliminated in tumor-bearing mice treated with trastuzumab plus pertuzumab plus C3E10 (Figure 4d).

## Disscusion

ErbB2 is proposed to heterodimerize with ligand-bound ErbBs using a largely domain II-mediated dimerization interface.^18^ In contrast, ErbB2 heterodimerization with ligand-free ErbBs may be mainly involved in domain IV contacts.^8^ The anti-ErbB2 antibodies trastuzumab and pertuzumab are directed against the ErbB2 heterodimerization interfaces.^14, 15, 18^ Pertuzumab binds to ErbB2 near the center of domain II^15^ and trastuzumab binds to the juxtamembrane region of domain IV.^14^ In this study, we describe a novel anti-ErbB2 antibody, 3E10, which binds to an epitope in domain III that appears to be located opposite to the dimerization interfaces in domain II and domain IV of ErbB2. Our data indicated that 3E10 blocked both ligand-independent and -dependent ErbB2 heterodimerization. Recently, *Fu et al.*^19^ also identified a domain III-specific antibody, hHERmAb-F0178C1. They found that hHERmAb-F0178C1 could block ligand-induced ErbB2/ErbB3 heterodimerization but their data did not show if hHERmAb-F0178C1 could inhibit ligand-dependent ErbB2/EGFR complex formation and ligand-independent ErbB2 heterodimerization. As the binding sites of hHERmAb-F0178C1 also appeared to be located opposite to the dimerization interfaces in domains II and IV, they hypothesize that ErbB2 might have activating membrane-associated ligands and that hHERmAb-F0178C1 might inhibit the heterodimerizition between ligand-bound ErbB2 and ligand-bound ErbB3 by disrupting the binding of ErbB2 with its ligands. However, our present study showed that the domain III-specific antibody 3E10 not only blocked ligand-dependent ErbB2 heterodimerization but also inhibited the formation of ligand-independent ErbB2/EGFR heterodimers and ligand-independent ErbB2/ErbB3 heterodimers. Moreover, no specific ligand for ErbB2 has yet been discovered. Therefore, we speculate that the 3E10 antibody may affect ErbB2 heterodimerization by causing major conformational changes of ErbB2.

Trastuzumab and pertuzumab mainly interfere with ligand-independent and ligand-induced ErbB2 heterodimerization, respectively.^8, 12^ Our present study has indicated that 3E10 inhibits ErbB2 heterodimerization via a mechanism that strikingly differs from trastuzumab and pertuzumab. Remarkably, 3E10 provides synergistic inhibition of ErbB2 heterodimerization and signaling in combination with either trastuzumab or pertuzumab. The combination of these three anti-ErbB2 antibodies that have complementary mechanisms of action appears to be an extremely potent ErbB2 heterodimerization blocker. Recently, pertuzumab in combination with trastuzumab has been approved for the treatment of patients with ErbB2-positive metastatic breast cancer. Compared with trastuzumab plus pertuzumab, the combination of trastuzumab, pertuzumab and 3E10 provides a more potent blockade of ErbB2 signaling and results in greater antitumor activity in ErbB2-overexpressing breast tumor models. Thus, it can be concluded that combinatorial treatment with these three mAbs may lead to a better therapeutic outcome for ErbB2-overexpressing breast cancer patients than trastuzumab plus pertuzumab.

In conclusion, our study identifies a new anti-ErbB2 antibody, which inhibits ErbB2 heterodimerization possibly by inducing major conformational changes of ErbB2 and provide synergistic inhibition of ErbB2 in combination with anti-ErbB2 antibodies directly disrupting heterodimerization. Importantly, the combination of trastuzumab, pertuzumab and 3E10 demonstrates a greater ability to inhibit ErbB2 signaling and breast cancer cell growth compared with trastuzumab plus pertuzumab, suggesting that it might be a promising treatment for ErbB2-overexpressing breast cancer.

## Materials and methods

### Cell lines and animals

The human breast cancer cell lines BT-474 and SK-BR-3 and the Chinese hamster ovary cell line CHO-K1 were obtained from the American Type Culture Collection (ATCC, Manassas, VA, USA). All the cell lines were authenticated twice by morphologic and isoenzyme analyses during the study period. Cell lines were routinely checked for contamination by mycoplasma using Hoechst staining and consistently found to be negative. Five-week-old female BALB/c nude mice were obtained from the Shanghai Experimental Animal Center of Chinese Academy of Sciences (Shanghai, China). All animals were treated in accordance with guidelines of the Committee on Animals of the Second Military Medical University.

### Hybridoma preparation

The extracellular domain of ErbB2 (ErbB2-ECD) was prepared as described previously,^15^ except that we used the pcDNA3.1(+)-expressing vector (Invitrogen, Waltham, MA, USA) and the FreeStyle 293 expression system (Invitrogen). Female BALB/c mice were repeatedly immunized with recombinant human ErbB2-ECD protein. Three days after the final immunization, spleens were collected and the splenocytes were fused to NS-1 mouse myeloma cells. The fused cells were cultured in hypoxanthine/aminopterin/thymidine medium. Culture supernatants from the resulting hybridomas were tested by ELISA for specific antibody reactivity to ErbB2-ECD. Antibody isotypes were determined by using a mouse mAb isotyping kit (Sigma, St Louis, MO, USA). Finally, the mouse anti-ErbB2 mAbs were purified by protein G affinity chromatography from hybridoma culture supernatants.

### Immunoprecipitation

The association of ErbB2 with ErbB3 cannot be detected using standard immunoprecipitation methods in the absence of ligand stimulation. In this study, the ligand-independent ErbB2-containing heterodimers were detected by using a reversible chemical crosslinking procedure described previously,^8^ with minor modifications. Briefly, cells were incubated with the indicated antibodies or peptides for 1 h at 37 °C. After washing twice with ice-cold HEPES/NaCl buffer (50 mM HEPES (pH 7.2), 150 mM NaCl), the cells were incubated with 2 mM 3,3′-dithiobis[sulfosuccinimidylpropionate] (DTSSP; Thermo Scientific, Rockford, IL, USA) dissolved in HEPES/NaCl buffer for 1 h at 4 °C. The cells were then washed three times with ice-cold 25 mM Tris (pH 7.1), 150 mM NaCl and lysed in NP-40 lysis buffer supplemented with protease and phosphatase inhibitors. For coimmunoprecipitation experiments, we incubated the total cell lysate with an agarose-conjugated anti-ErbB2 monoclonal antibody (sc-7301 AC; Santa Cruz Biotechnology, Santa Cruz, CA, USA) overnight at 4 °C. The precipitated proteins were subjected to sodium dodecyl sulfate polyacrylamide gel electrophoresis followed by western blot analysis with antibodies specific for EGFR (sc-03; Santa Cruz Biotechnology), ErbB2 (sc-7301; Santa Cruz Biotechnology) or ErbB3 (sc-285; Santa Cruz Biotechnology).

The formation of ligand-induced ErbB2-containing heterodimers was assayed by the method described previously,^12^ with slight modifications. Briefly, the cells were starved overnight in growth medium without serum and then incubated with the indicated antibodies or peptides for 1 h at 37 °C. Recombinant human EGF (R&D Systems, Minneapolis, MN, USA) and HRG (R&D Systems) were added at a final concentration of 5 and 1 nM, respectively. EGF is a ligand for EGFR and HRG is a ligand for ErbB3 and ErbB4. After an additional 10 min incubation, the cells were washed three times and lysed in NP-40 lysis buffer. The coimmunoprecipitation experiments were then performed as described above.

### Immunoblotting

Western blotting was performed to examine the effects of anti-ErbB2 antibodies on the phosphorylation of ErbBs, MAPK and AKT. In our previous study, we have used western blot analysis to determine the expression levels of EGFR, phospho-EGFR, ErbB2, phospho-ErbB2, ErbB3, phospho-ErbB3 in several breast cancer cell lines after treatment with different anti-ErbB2 antibodies for 1 h.^20^ Our data showed that neither of the anti-ErbB2 antibodies affected the expression levels of EGFR, ErbB2 and ErbB3.^20^ Consistent with our results, other groups also demonstrated that treatment with the anti-ErbB2 antibody did not affect the expression levels of ErbBs.^8, 21^ Therefore, in western blotting assay of the present study, we only used glyceraldehyde-3-phosphate dehydrogenase (GAPDH) as a control and did not determine the expression levels of ErbBs. Briefly, cells were incubated with the indicated antibodies in serum-free medium for 1 h at 37 °C. The cells were then treated with EGF (5 nM) or HRG (1 nM) or not treated for 15 min. After washing, the cells were lysed in sodium dodecyl sulfate lysis buffer and the cell lysates were subjected to sodium dodecyl sulfate polyacrylamide gel electrophoresis and immunoblotted with antibodies against phospho-EGFR-Tyr1068 (2236; Cell Signaling, Danvers, MA, USA), phospho-ErbB2-Tyr1221/1222 (2243; Cell Signaling), phospho-ErbB3-Tyr1289 (4791; Cell Signaling), phospho-AKT-Ser473 (4060; Cell Signaling) or phospho-p44/42 MAPK-Thr202/Tyr204 (9106; Cell Signaling).

### Cell proliferation assay

Cells were incubated with recombinant anti-ErbB2 mAbs for 2 h, followed by the addition of ErbB ligands or not. Recombinant human EGF and HRG were added at a final concentration of 5 and 1 nM, respectively. After an additional 4-day incubation, cell proliferation was determined by CellTiter 96 AQueous One Solution Cell Proliferation Assay (MTS assay) kit (Promega, Madison, WI, USA). All measurements were performed in triplicate.

### *In Vivo* therapy study

Female BALB/c nude mice were implanted with 0.72 mg 60-day release 17β-estradiol pellets (Innovative Research of America, Sarasota, FL, USA). After 6 days, 1 × 10^7^ BT-474 cells were injected into the mammary fat pad in a 1:1 PBS:matrigel suspension (BD matrigel; BD Biosciences, San Jose, CA, USA). When tumor volumes reached an average of about 100 mm^3^, the mice were randomly divided into groups of 10 mice each. Treatments consisted of twice weekly intravenous injection of different anti-ErbB2 mAbs for four consecutive weeks. Control mice were given vehicle (IgG) alone. Tumors were measured with digital calipers, and tumor volumes were calculated by the formula: volume=length × (width)^2^/2. Animal procedures were performed under an approved protocol.

### Phage display peptide library screening

The PhD-7 phage display peptide library kit was purchased from New England BioLabs (Beverley, MA, USA). Biopanning of PhD-7 phage display peptide library with mouse anti-ErbB2 mAbs was performed according to the manufacturer's instructions.

### Phage ELISA

ELISA screening of phage clones was performed as previously reported.^22^ Briefly, 100 μl of supernatant containing amplified particles from each phage clone were added to 96-well plates precoated with mouse anti-ErbB2 mAbs. After incubation for 2 h at room temperature, detection was carried out with horseradish peroxidase-conjugated anti-phage M13 monoclonal antibody (GE Healthcare, Marlborough, MA, USA). Finally, positive phage clones were subjected to DNA sequence analysis.

### Mutation analysis

Mutations were introduced by overlapping PCR into the extracellular domain gene of ErbB2, and the ErbB2-ECD mutant proteins were expressed and purified using the same procedure as for the wild-type ErbB2-ECD protein. An amount of 2 μg/ml of wild-type ErbB2-ECD (WT) or ErbB2-ECD mutants were added to 96-well plates precoated with 5 μg/ml of trastuzumab, followed by incubation at 37 °C for 1 h. The plates were washed, and different concentrations of 3E10 or the control mouse antibody 9F12 were added to each well and incubated at 37 °C for 1 h. After washing, horseradish peroxidase-conjugated goat polyclonal secondary antibody to mouse IgG-H&L was added and the plates were further incubated for 1 h at 37 °C. Finally, TMB was added as a substrate and the absorbance was read at 450 nm.

### Competitive binding assay

Cells at 1 × 10^6^ cells/ml were incubated with a subsaturating concentration of the indicated Alexa Fluor 488-conjugated anti-ErbB2 mAbs and increasing concentrations of purified competing antibodies for 1 h at 4 °C. Then, the cells were washed and analyzed by flow cytometry using a FACScan flow cytometer (Becton Dickinson, San Jose, CA, USA).

### Statistical analysis

Statistical analysis was performed by Student's unpaired *t-*test to identify significant differences unless otherwise indicated. Differences were considered significant at *P*<0.05. Statistical analyses were performed in GraphPad Prism 5 (GraphPad Software, La Jolla, CA, USA).
